# Physico-Chemical and Sensory Profiles of Enriched Linz Biscuits

**DOI:** 10.3390/foods10040771

**Published:** 2021-04-04

**Authors:** Zuzana Hlaváčová, Eva Ivanišová, Ľuboš Harangozo, Ana Petrović, Denisa Kušteková, Branislav Gálik, Peter Hlaváč, Monika Božiková, Vlasta Vozárová

**Affiliations:** 1 Department of Physics, Faculty of Engineering, Slovak University of Agriculture in Nitra, Trieda A. Hlinku 2, SK-949 76 Nitra, Slovakia; ana.petrovic@uniag.sk (A.P.); peter.hlavac@uniag.sk (P.H.); monika.bozikova@uniag.sk (M.B.); vlasta.vozarova@uniag.sk (V.V.); 2 Department of Technology and Quality of Plant Products, Faculty of Biotechnology and Food Sciences, Slovak University of Agriculture in Nitra, Trieda A. Hlinku 2, SK-949 76 Nitra, Slovakia; eva.ivanisova@uniag.sk (E.I.); Kustekova.deniska@gmail.com (D.K.); 3 Department of Chemistry, Faculty of Biotechnology and Food Sciences, Slovak University of Agriculture in Nitra, Trieda A. Hlinku 2, SK-949 76 Nitra, Slovakia; lubos.harangozo@uniag.sk; 4 Department of Animal Nutrition, Faculty of Agrobiology and Food Resources, Slovak University of Agriculture in Nitra, Trieda A. Hlinku 2, SK-949 76 Nitra, Slovakia; branislav.galik@uniag.sk

**Keywords:** enriched biscuits, heat of combustion, electrical properties, chemical composition, sensory properties

## Abstract

The aim of the present study was to determine the physico-chemical properties (dry matter content, combustion heat, electrical properties, total protein, ash, fat and crude fibre contents, selected amino acids, and trace elements), antioxidant content, and sensory profile of Linz biscuits. They were enriched by the addition of powdered carrot, nettle leaves and elderberry fruit, which is 3% of the product. For comparison of results, a control variant without the addition of these components was also prepared. The enriched biscuits showed slightly higher total ash and crude fibre contents in comparison to the control samples. Results for the antioxidant activity and total polyphenol, flavonoid, and phenolic acid contents of the enriched biscuits were higher in all observed parameters than in the control sample with the best results obtained for Linz biscuits enriched with elderberry and nettle powder. In enriched biscuits, higher contents of iron, zinc, and manganese were measured, especially in biscuits with nettle. Linz biscuits with nettle had higher combustion heat values than control samples; the other two sample types had lower values. We found that the resistance, capacitance, and relative permittivity of the enriched biscuits decreased with frequency according to the power regression function. On the contrary, the conductivity increased with an increasing frequency. Electrical properties were mainly influenced by the water content but also by added components.

## 1. Introduction

Knowledge of physico-chemical properties of agricultural products is essential to develop methods for food material evaluation. The food industry is developing new products using functional foods and ingredients, creating new value-added foods, because of consumers’ demand for healthier food [1]. This development has the advantage of allowing food manufacturers to add extra value to products that the consumer is already familiar with. The main factors that have to be considered are the variations affecting the processing conditions, the sensory properties, and the nutritional value of the final product [1,2]. Some studies have addressed the impacts of various additives on the quality of food, e.g., as value-added compounds. Almeida et al. [3] described the using of hardy kiwifruit (*Actinidia arguta*) leaves, Różyło et al. [4] investigated carob (*Ceratonia siliqua* L.) fibres, and Pasqualone et al. [1] studied grape marc extract.

Biscuits, a low cost, processed food, are widely consumed due to their numerous benefits: good price compared with conventional snack items, easy to consume at home or during travel, easily available in a wide variety of shapes, sizes, tastes, and packs, and suitable for all age groups [5]. They cannot, however, be regarded as a healthy snack food because they usually contain high levels of easily digested carbohydrates and fats, generally low levels of biologically active compounds, and only modest levels of protein, as they are usually made from flour, butter, and sugar. Recent trends suggest that people are becoming aware of the food they consume, and they are also aware of the benefits of consuming nutritious biscuits [6]. Recently, increasing consumer demand for healthier foods has triggered the development of biscuits made with natural ingredients exhibiting functional properties and providing specific health benefits [7]. Carrot (*Daucus carota* L.) is a very good source of all vitamins, minerals, and dietary fibre. Dried carrot powder contains carotene, ascorbic acid, cellulose, pectin, hemicellulose, and lignin [8]. Stinging nettle (*Urtica dioica* L.) is a ubiquitous herb that is available in large part of the world. Stinging nettle leaves contain mainly vitamins A and C, calcium, iron, and sodium and have a rich fatty acid profile [9]. Elderberry (*Sambucus nigra* L.) grows in Europe and Western Asia (with black berries). The biological value of the fruit comes from its high levels of vitamins, minerals, pectin, colour, cellulose, dietetic fibre, sugars, and organic acids and its low energetic value. Because of all this, consuming fruit and fruit products is favourable in everyday life [10].

Food is heated for many reasons such as to make food more palatable and easier to digest, to add flavour, to make cooked food last longer (by killing microbes), and to aid in the process of converting starches into simple sugars [11]. In the praxis, for food, the heat of combustion is measured and, on this basis, the amount of energy that the organism consumes can be calculated. The heat of combustion *Q_c_* (J) is the amount of heat that is released from the substance by perfect combustion or another degradation process at a constant temperature. The specific heat of combustion *q_c_* (J/kg) is the combustion heat calculated per unit of mass. We can use Equation (1).

(1) $$q_{c}=\frac{Q_{c}}{m}$$

where *m* is the mass (kg). The specific heat of combustion can also be calculated per unit of volume or per unit of substance. In photosynthesis, plants use sunlight to convert carbon dioxide and water into the simple sugar glucose and oxygen. This energy is stored in the chemical bonds that become bioavailable when eaten by other organisms [11].

Electrical properties are used in many areas of human activity. They are also important for the sustainable control of food preparation and processing. Measurement of the electrical properties of various agricultural products and foods is used to determine different parameters, mainly the moisture content, and also chemical components, the germinability of seeds and grains, and the resistance of fruits to frost [12,13]. Through the use of electrical resistance or conductance, RF capacitance measurements for dielectric heating parameter determination and microwave measurements of dielectric properties for quality sensing, e.g., of fruits, vegetable or milk, have been studied for many years [14,15,16].

The aim of this research was to analyse physical, chemical, antioxidant, and organoleptic parameters of Linz biscuits with and without the addition of 3% carrot, nettle, and elderberry fruit powder. We chose to add 3% based on previous studies [17] in which plant additions of 1%, 3%, and 5% to Linz biscuits and pasta were tested, and 3% was the maximum addition amount that was acceptable from a sensory point of view.

## 2. Materials and Methods

The root of carrot (*Daucus carrota* L.) harvested in autumn (September 2019), nettle leaves (*Urtica dioica* L.) harvested in spring (May 2019) and elderberry fruits (*Sambucus nigra* L.) harvested in autumn (September 2019) in Liešťany village located in Central Slovakia at an altitude of 333 m a.s.l. were used in this study. The plant materials were botanically identified at the Department of Technology and Quality of Plant Products of the Slovak University of Agriculture in Nitra. Roots, leaves, and fruits were dried in an oven cabinet (Binder M 115, Germany) at 40 °C for 48 h and homogenized to powder in a mortar.

The sweet Linz biscuits were prepared according to the AACC method [18] with slight modifications. The ingredients were purchased at the local market and included wheat flour (200 g), sugar (75 g), salt (0.01 g), sodium bicarbonate (1 g), butter (125 g), vanilla (0.025 g), egg yolk (15 g), and lemon peel (0.1 g). Preparation of biscuits was carried out using wheat flour (control) and wheat flour samples enriched with 3% carrot, nettle, and elderberry fruit fibre powder. Each type of biscuit was prepared separately. Shortening and creaming techniques were used. The biscuits were baked at 120 °C for 20 min (oven Miwe condo, Germany). After cooling for 30 min, the biscuits were packed and evaluated for their physical, chemical, antioxidant, and sensory characteristics.

The heat of combustion and electrical properties were measured on the Department of Physics. A calorimeter IKA C 5000 (IKA Works, Inc., Wilmington, NC, USA) was used to measure the heat of combustion. The calorimetric system of this apparatus used for solid and liquid substances allows both adiabatic and isoperibolic methods to be used. It also allows for the selection of other measurement methods such as dynamic methods. We used the adiabatic method which is more convenient for the measurement of loose samples. The energy value of a food is the amount of heat that will be released when the food is fully burned. The heat of combustion was measured twice, and the average value was calculated for all samples.

Electrical properties are properties that characterize the transport of charge carriers in the material or the propagation of electromagnetic waves in the material. Low-frequency electrical properties of biscuits were measured with a GoodWill Instek LCR meter 821(GW Instrument Co., Ltd., Taipei, Taiwan) instrument at different frequencies using a four-electrode (tetra polar) system. We measured the capacitance and resistance. Two sample holders were used: the first was a cylindrical sensor with parallel electrodes and the second sensor was a coaxial capacitor. The cylindrical sensor was used for resistance measurement, and it had the following parameters: an electrode diameter of 37.8 mm and an electrode spacing of 49.2 mm. The upper electrode had a spring such that the sample was uniformly compressed in the sensor. We used a second sensor that was coaxial for the capacitance measurements. The radius of the capacitor’s outer electrode was 33 mm, the radius of the inner electrode was 8 mm and length of capacitor was 60 mm. We used homogenized Linz biscuit samples from the Department of Technology and Quality of Plant Products for measurements. The samples were homogenized in the mortar, so that the average particle size was 0.85 mm. This kind of sample was also used during the combustion heat measurement. For the first sensor, the compression of the sample was ensured by a spring, and the sample always had the same height. The compaction of the sample in the second sensor was ensured by shaking. Each value of resistance and capacitance was measured three times at all analysed frequencies in the range from 0.1 to 200 kHz. The average values were computed from these ones and standard deviations were calculated. The conductivity *σ* (S/m) and relative permittivity *ε*_r_ were calculated using Equations (2) and (3), respectively.

(2) $$\sigma =\frac{\ell }{R\;S}$$

where *ℓ* is the electrodes spacing in m, *R* is the resistance in Ω, and *S* is the electrode area in m^2^);

(3) $$\varepsilon_{r}=\frac{C\;\ln\frac{r_{2}}{r_{1}}}{2\;\pi \;\varepsilon_{0}\;\ell }$$

where *C* is the capacitance in F, *r*_1_ is the radius of the inner electrode in m, *r*_2_ is the radius of the outer electrode in m, *ε*_0_ is the relative permittivity of the vacuum in 8.854 × 10^−12^ F/m), and *ℓ* is the sample height in the capacitor in m.

For chemical evaluation, the dry matter, ash, and protein were determined in accordance with AACC standard 08-01 [19]. The nitrogen content was measured by the semi-micro Kjeldahl method. Nitrogen was converted to protein using a factor of 5.7. The crude fibre content was evaluated with the Ancom200 Fiber Analyzer (Ancom Technology, Fairport, NY, USA) according to the producer method. The fat content was evaluated with the Ancom XT15 Fat Extractor (Ancom Technology, Fairport, NY, USA) following the instructions of the manufacturer. The amino acid composition was determined by ion-exchange chromatography with a strong cation ion-exchanger and a sodium–citrate elution buffer system followed by postcolumn derivatization with ninhydrin and spectrophotometric detection, according to the standard protocol of the manufacturer of the amino acid analyser (Ingos, Prague, Czech Republic). For calibration of the amino acid analyser, the amino acid standard solution was used. Tryptophan was not determined, as it is destroyed during acid hydrolysis, and asparagine and glutamine change to aspartic acid and glutamic acid and are determined in these forms. The analysis of trace elements (Cd, Pb, Cu, Zn, Co, Cr, Ni, Mn, and Fe) was performed with an atomic absorption spectrometer Varian model AA 240 FS equipped with a D2 lamp background correction system using an air-acetylene flame (air 13.5 L/min, acetylene 2.0 L/min) (Varian, Ltd., Mulgrave, Australia). The measured results were compared with the multielement standard for GF AAS (CertiPUR^®^, Merck, Germany).

For antioxidant evaluation, the reducing power and radical scavenging activity DPPH (2,2-diphenyl-1-picrylhydrazyl) method was used. The reducing power was determined by the phosphomolybdenum method of Prieto et al. [20] with slight modifications. Radical scavenging activity was determined using the DPPH method according to Sánchéz-Moreno et al. [21] with slight modifications. The total polyphenol content was determined spectrophotometrically by a method using the Folin–Ciocalteu reagent according to Singleton and Rossi [22]; the total flavonoid content was determined spectrophotometrically with the aluminium–chloride method according to Willett [23]; and the total phenolic acid content was determined spectrophotometrically with Arnova reagent according to Farmakopea Polska [24].

The organoleptic properties of prepared biscuits were determined by a taste panel of 20 evaluators (average consumers). The panellists were asked to evaluate the flavour of the product (overall), flavour (intensity), foreign flavour (presence), taste (overall), taste (intensity), aftertaste, and overall acceptability. All parameters were compared with a control sample without the enrichment of powder. The ratings were on a 9-point hedonic scale ranging from 9 (like extremely) to 1 (dislike extremely) for the flavour of the product (overall), flavour (intensity), taste (overall), taste (intensity), and overall acceptability and from 9 (presence) to 1 (non-presence) for the foreign flavour (presence) and aftertaste.

All experiments were carried out in triplicate, and the results are reported as the mean of the replicate determinations with standard deviations. The experimental data were subjected to an analysis of variance (ANOVA) using Duncan’s test to determine the level of significance between experimental groups at the confidence level of 0.05 using SAS software [25].

## 3. Results

The results are presented in the following order: chemical composition, presence of antioxidants, sensory analysis, and physical properties of biscuits.

### 3.1. Chemical Composition

The control sample of Linz biscuits contained lower levels of ash and crude fibre. Dry matter (~95%), crude protein (~6%), and fat content (~25%) were similar in all kinds of biscuit. The dry matter, crude protein, ash, fat, and crude fibre contents are shown in Table 1.

Linz biscuits enriched with nettle leaf powder contained the highest total ash value, in which iron had the highest content (Table 2) (1.23 mg per 100 g) in comparison with the control sample and the sample with carrot and elderberry. In this sample, higher amounts of manganese and zinc were found. The presence of hazardous elements (lead, chrome, cadmium and cobalt) was determined only in trace amounts, which is in accordance with the Slovak legislative [26]. The content of total protein was slightly lower than in the control variant, but the results for the amino acid composition (Table 3) were interesting as the amounts of proline, valine, and phenylalanine were slightly higher than in the control sample.

Similar findings were also reported by Adhikari et al. [9], who analysed pure powder from nettle leaves and found a high level of essential amino acids, which was higher than for wheat and barley flour. In our study, we applied 3% nettle leaf powder, but it is possible that a higher amount could markedly increase the protein content as well as the amino acid composition. In enriched biscuits, it is very important to find an optimal percentage of enrichment. A high percentage can increase the nutritional value of products but can negatively influence sensory profiles. For this reason, it is important to find an optimal value that is acceptable for the consumer and also good from a nutritional point of view. This optimal value in our case was 3% in the case of addition [17].

Linz biscuits enriched with carrot powder had higher contents of ash, fat, and crude fibre in comparison to the control sample. They also had trace element and amino acid compositions that were very similar to the control sample. The presence of hazardous elements (lead, chrome, nickel, and cobalt) was determined only in trace amounts, which is in accordance to Slovak legislative [26]. Gayas et al. [27] published similar results for the total ash and protein contents in biscuits with carrot pomace, whereas the total ash content was slightly higher in biscuits with carrot in comparison to in the control samples. The total protein content decreased in their study with an increase in the level of carrot pomace powder, because carrot pomace powder has a lower protein content than wheat flour.

Linz biscuits enriched with elderberry fruit powder contained a higher level of total ash in comparison to the control samples. In trace elements, higher contents of iron and zinc were found (Table 2). The presence of hazardous elements (lead, chrome, nickel and cobalt) was determined only in trace amounts, which is in accordance with Slovak legislative [26]. In this sample, the highest content of crude fibre was also determined. The addition of this kind of powder had no influence on the amino acid composition. The results were similar to those of control sample, although the amount of threonine was slightly higher (Table 3).

### 3.2. Antioxidant Activity

Antioxidant activity (Table 4) was tested using two different methods—DPPH and reducing power. Linz biscuits enriched with nettle leaf powder and elderberry fruit powder showed the highest values tested by these methods. In these samples, there was also a higher level of total polyphenols as well as total flavonoids and phenolic acids compared to the control sample.

The lowest antioxidant activity and total polyphenol, flavonoid, and phenolic acid contents in enriched biscuits were detected in the samples with carrot, but the activity and phenolic content were still higher than in the control samples. Enrichment of biscuits with various plant materials is effective from this point of view because it can increase antioxidant activity as well as the content of biologically active compounds. In this study, a statistically strong correlation (*p* ≤ 0.05) was also observed between antioxidant activity by DPPH and the total flavonoid content (r = 0.969) and between the reducing power and total polyphenol (r = 0.961) and phenolic acid contents (r = 0.998). An increase in the biological activity of enriched biscuits was also confirmed by Bolanho et al. [28], who compared control samples with biscuits enriched with Spirulina platensis powder and reported a 37% increase in antioxidant activity and a 64% increase in the phenolic composition in enriched biscuits. An increase in the total polyphenol content was also confirmed by Molnar et al. [29], who prepared biscuits enriched with black currant and jostaberry powder and compared these biscuits with control samples without any additions. In a study by Ahmed et al. [30], an increase in the nutritional value of biscuits enriched with Cyperus esculentus L. powder in comparison to control samples was also confirmed. These authors detected an increase not only in the total polyphenol content but also in crude fiber, zinc, and iron. Our results are in accordance with these findings.

### 3.3. Sensory Analysis

**Figure 1:**
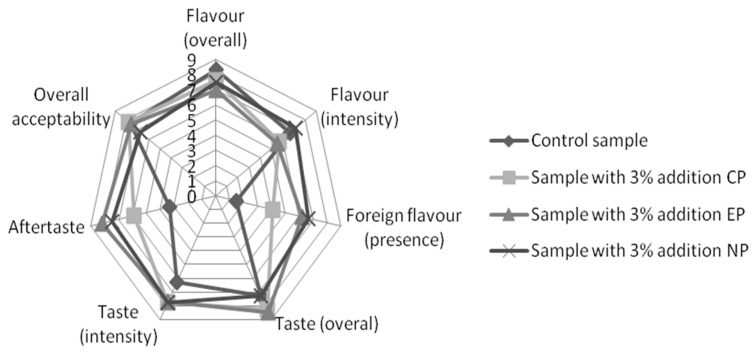
Results for the sensory characteristics of prepared biscuits (sum of all evaluations)—CP (carrot powder), EP (elderberry powder), NP (nettle powder).

The results of the taste, flavour, and overall acceptability of biscuits showed no strong differences in the sensory evaluation (Figure 1). The aftertaste characteristics varied significantly, and some evaluators described a “grassy taste” for biscuits with 3% nettle powder, while another noted a pleasant spice and fruity aftertaste for biscuits with 3% elderberry powder. Generally, in all tested biscuits harmonic, pleasant characteristics were detected with the biscuits enriched with 3% carrot powder having the best evaluation.

Biscuits with the addition of 6% of carrot pomace powder had the highest scores in a study by Mounika and Maloo [31]. In a study by Alam et al. [32], the received the biscuits with 1% herbal addition received the highest evaluation. The organoleptic properties of enriched biscuits are important because this may improve a consumer’s attitude to products with good nutritional value, so the acceptable amount of additives for a product must be identified. From a sensorial point of view, we generally obtained good acceptance for the Linz biscuits with 3% admixtures in our study.

### 3.4. Physical Properties

During measurement of the specific heat of combustion, we found that there were no large differences between the two repetitions. It was sufficient to determine the average value from two measurements. In Table 5, we can see that Linz biscuits with nettle had higher values of combustion heat than the control sample, and the other two samples had lower values. This result was affected by the chemical compositions of the studied samples.

The fat content was associated with the biggest increase in the combustion heat value [11]. Although the sample with elderberry had the highest fat content, the protein content was lower and, therefore, the sample had a lower specific combustion heat. In the literature, there is insufficient information about the combustion heat of foods.

**Figure 2:**
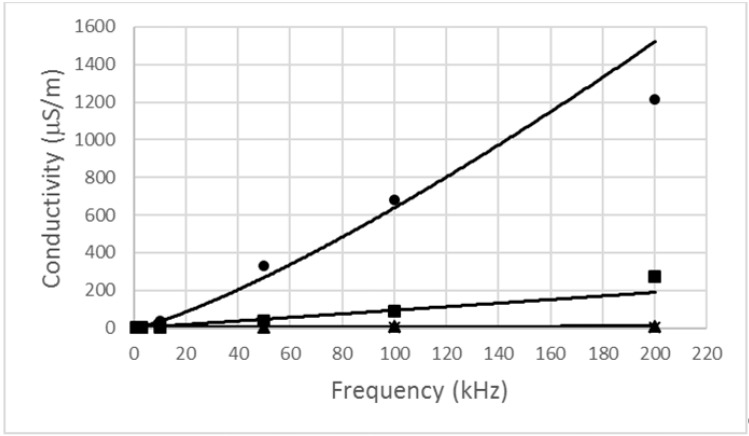
Conductivity of biscuit samples versus frequency (*—control sample, ▲—with nettle, ■—with carrot, •—with elderberry).

We found that the conductivity of the four biscuit samples increased as the frequency increased (Figure 2).

The power model is suitable for the description of this dependence. It is shown in Equation (4).

(4) $$\sigma =\sigma_{0}{\left(\frac{f}{f_{0}}\right)}^{k}$$

where *σ* is the conductivity in S/m, *σ*_0_ is the reference value of conductivity in S/m, *f* is the frequency in Hz, *f*_0_ is 1 kHz, and *k* is a constant.

From Figure 2, it is evident that the sample with elderberries had the highest conductivity values at all frequencies, and the lowest values were for the control biscuit samples and samples with nettle. The conductivity of food is significantly influenced by the moisture content. The moisture content wet basis was determined on the basis of the dry matter content (Table 1) as a supplement up to 100%. The differences were low between samples. The moisture content was highest for biscuits with elderberries and lowest for biscuit with nettle. However, again, it has been confirmed that the moisture content affects the electrical properties of materials similarly, as shown, e.g., in papers by Justicia et al. [13] and Nelson and Trabelsi [14]. The conductivity of biscuits is also influenced by the added components.

On the contrary, the resistance decreases with frequency according to a power regression function similar to Equation (4). The reason for this is that the conductivity is inversely proportional to the resistance (Equation (2)).

**Figure 3:**
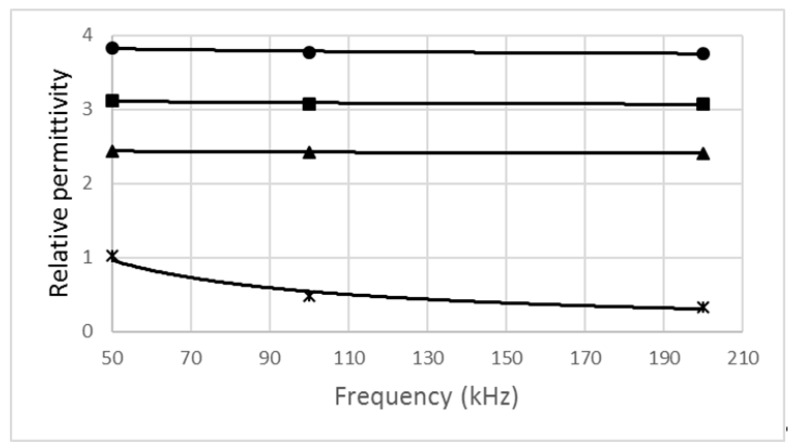
Relative permittivity of biscuit samples versus frequency (*—control sample, ▲—with nettle, ■—with carrot, •—with elderberry).

Figure 3 represents a relation between the relative permittivity of biscuit samples and the frequency.

We chose the frequency interval from 50 to 200 kHz, which is useful for comparing dependencies for various biscuits. The relative permittivity of the biscuits decreased with the frequency. In this case, we can also use a power function similar to the model (Equation (4)). All enriched biscuits had higher relative permittivities than the control sample. The highest values were obtained for biscuits with elderberry fruit powder. Additionally, in this case, the influence of the moisture content wet basis was manifested. Electrical properties were influenced by components added to biscuits. The power model is also valid for capacitance, because, according to Equation (3), the relative permittivity is proportional to the capacitance, which also decreases with frequency.

We used the power model to describe the dependency of the electrical properties on the frequency. All dependencies had very high coefficients of determination in the range of 0.8085–0.9938, so the relations were very well described by the power function. For example, Guo et al. [33] used the relative permittivity measured at higher frequencies to identify the sugar content and water content of the different types of honey and the moisture content in chickpea flour [34], and Łuczycka et al. [35] found a relationship between dielectric properties and the oat meal admixture in wheat flour. In the literature, information on the electrical properties of biscuits is not found. Our measurements indicate that samples of biscuits must be considered as complex objects. They are heterogeneous, multi-component semiconductors or dielectrics. We found that value-added biscuits have different electrical properties. On the basis of the relative permittivity, we could distinguish the admixture added to biscuits. Further research in this area is needed to clarify the impacts of value-added components in biscuits.

## 4. Conclusions

Sweet Linz biscuits were prepared using the AACC method. The preparation of biscuits was carried out using wheat flour (control) and wheat flour samples enriched with 3% carrot, nettle, and elderberry fruit powder fibre. The addition of these materials to biscuits enhanced the nutritional value of the product by increasing the ash and crude fibre contents. In these biscuits, strong increases in antioxidant activity as well as in the total polyphenol, flavonoid, and phenolic acid contents were observed. Moreover, the addition of powder improved the sensory characteristics of the product with the best overall acceptability found for biscuits with 3% carrot powder. Therefore, the application of carrot, nettle, and elderberry powder in the production of Linz biscuits is recommended to target the nutritional and sensorial properties of this bakery product.

From a physical point of view, we can conclude that Linz biscuits with nettle have higher values of combustion heat than the control samples. The other two samples had lower values. The resistance, capacitance, and relative permittivity of enriched biscuits decreased with the frequency according to the power regression function in this frequency range. On the contrary, the conductivity increased as the frequency increased. All enriched biscuits had higher levels of conductivity and relative permittivity than the control samples. The highest values were obtained for biscuits with powder from elderberry fruits. Electrical properties are mainly influenced by the water content but also by added components. We found out that a correlation exists between electrical properties and the type of admixture added to the biscuits. Electric properties are easily measurable and can be used to determine other characteristics of value-added foods. On the basis of the relative permittivity, we were able to distinguish the component added to the biscuits. Further research in this area is needed to clarify the impacts of value-added components on the biscuit.

## Figures and Tables

**Table 1:** Some chemical component contents in prepared biscuits.

Sample	DMC (%)	CP (%)	AC (%)	FC (%)	CFC (%)
control	95.00 ± 0.23 ^c^	6.37 ± 0.08 ^a^	1.03 ± 0.09 ^c^	24.18 ± 0.12 ^d^	0.48 ± 0.01 ^c^
with nettle	95.76 ± 0.18 ^a^	5.57 ± 0.18 ^c^	1.48 ± 0.04 ^a^	25.15 ± 0.18 ^c^	0.51 ± 0.03 ^ab^
with carrot	95.61 ± 0.32 ^b^	6.03 ± 1.04 ^b^	1.19 ± 0.04 ^b^	25.25 ± 0.24 ^b^	0.50 ± 0.04 ^bc^
with elderberry	95.47 ± 0.29 ^b^	5.49 ± 0.16 ^c^	1.20 ± 0.05 ^b^	27.52 ± 0.16 ^a^	0.52 ± 0.04 ^a^

mean value ± standard deviation; DMC (dry matter content); CP (crude protein); AC (ash content); FC (fat content); CFC (crude fibre content); different letters in a column denote mean values that statistically differ one from another.

**Table 2:** Trace element contents in prepared biscuits.

Parameter (mg Per 100 g)	Control	with Nettle	with Carrot	with Elderberry
Copper (Cu)	0.12 ± 0.01 ^a^	0.12 ± 0.01 ^a^	0.11 ± 0.02 ^a^	0.12 ± 0.01 ^a^
Manganese (Mn)	0.36 ± 0.02 ^b^	0.51 ± 0.02 ^a^	0.39 ± 0.02 ^b^	0.38 ± 0.02 ^b^
Iron (Fe)	0.97 ± 0.05 ^c^	1.23 ± 0.04 ^a^	0.94 ± 0.05 ^d^	1.14 ± 0.03 ^b^
Zinc (Zn)	0.54 ± 0.02 ^c^	0.62 ± 0.02 ^a^	0.52 ± 0.03 ^d^	0.58 ± 0.02 ^b^
Chrome (Cr)	0.03 ± 0.01 ^a^	0.04 ± 0.01 ^a^	0.04 ± 0.01 ^a^	0.04 ± 0.01 ^a^
Nickel (Ni)	0.04 ± 0.01 ^b^	nd	0.05 ± 0.03 ^a^	0.03 ± 0.02 ^c^
Cobalt (Co)	0.04 ± 0.02 ^a^	0.02 ± 0.01 ^b^	0.02 ± 0.01 ^b^	0.04 ± 0.01 ^a^
Lead (Pb)	0.06 ± 0.02 ^a^	0.03 ± 0.02 ^c^	0.05 ± 0.02 ^b^	0.03 ± 0.02 ^c^
Cadmium (Cd)	nd	nd	0.06 ± 0.03 ^a^	nd

mean ± standard deviation; nd (not detected); different letters in a column denote mean values that statistically differ one from another.

**Table 3:** Amino acid composition of prepared biscuits.

Parameter (μg Per 100 g)	Control	with Nettle	with Carrot	with Elderberry
Aspartic acid (Asp)	0.62 ± 0.21 ^b^	0.69 ± 0.11 ^a^	0.53 ± 0.11 ^d^	0.56 ± 0.09 ^c^
Threonine (Thr)	0.19 ± 0.02 ^c^	0.21 ± 0.05 ^b^	0.19 ± 0.02 ^c^	0.69 ± 0.11 ^a^
Serine (Ser)	0.34 ± 0.01 ^c^	0.36 ± 0.08 ^b^	0.34 ± 0.08 ^c^	0.39 ± 0.05 ^a^
Glutamic acid (Glu)	1.79 ± 0.14 ^b^	1.81 ± 0.21 ^a^	1.78 ± 0.14 ^b^	1.79 ± 0.11 ^b^
Proline (Pro)	0.28 ± 0.12 ^b^	0.65 ± 0.11 ^a^	0.29 ± 0.11 ^b^	0.29 ± 0.03 ^b^
Glycine (Gly)	0.23 ± 0.11 ^a^	0.23 ± 0.05 ^a^	0.21 ± 0.04 ^ab^	0.21 ± 0.02 ^b^
Alanine (Ala)	0.19 ± 0.06 ^a^	0.19 ± 0.01 ^a^	0.16 ± 0.02 ^b^	0.16 ± 0.01 ^b^
Valine (Val)	0.24 ± 0.09 ^b^	0.27 ± 0.04 ^a^	0.24 ± 0.04 ^b^	0.24 ± 0.13 ^b^
Isoleucine (Ile)	0.21 ± 0.07 ^b^	0.23 ± 0.13 ^a^	0.21 ± 0.02 ^b^	0.21 ± 0.05 ^b^
Leucine (Leu)	0.45 ± 0.12 ^b^	0.48 ± 0.09 ^a^	0.44 ± 0.11 ^c^	0.44 ± 0.04 ^c^
Tyrosine (Tyr)	0.32 ± 0.14 ^a^	0.29 ± 0.03 ^c^	0.31 ± 0.08 ^b^	0.31 ± 0.11 ^b^
Phenylalanine (Phe)	0.34 ± 0.05 ^b^	0.38 ± 0.02 ^a^	0.32 ± 0.05 ^c^	0.32 ± 0.03 ^c^
Histidine (His)	0.19 ± 0.11 ^b^	0.16 ± 0.06 ^d^	0.17 ± 0.03 ^c^	0.71 ± 0.05 ^a^
Lysine (Lys)	0.19 ± 0.02 ^a^	0.19 ± 0.11 ^a^	0.19 ± 0.02 ^a^	0.19 ± 0.02 ^a^
Arginine (Arg)	0.46 ± 0.14 ^a^	0.32 ± 0.08 ^b^	0.29 ± 0.06 ^c^	0.29 ± 0.14 ^c^

mean ± standard deviation; different letters in a column denote mean values that statistically differ one from another.

**Table 4:** Results of the antioxidant evaluation of prepared biscuits.

Parameter	Control	with Nettle	with Carrot	with Elderberry
DPPH (mg TEAC g^−^^1^)	1.02 ± 0.08 ^d^	1.57 ± 0.07 ^b^	1.09 ± 0.02 ^c^	1.47 ± 0.01 ^a^
RP (mg TEAC g^−^^1^)	51.27 ± 0.62 ^d^	68.26 ± 2.09 ^b^	61.16 ± 0.97 ^c^	87.21 ± 4.84 ^a^
TPC (mg GAE g^−^^1^)	0.09 ± 0.01 ^d^	0.31 ± 0.04 ^b^	0.27 ± 0.01 ^c^	1.47 ± 0.03 ^a^
TFC (mg QE g^−1^)	nd	0.08 ± 0.01 ^a^	0.03 ± 0.01 ^c^	0.05 ± 0.02 ^b^
TPAC (mg CAE g^−1^)	nd	0.03 ± 0.01 ^b^	0.02 ± 0.01 ^c^	0.07 ± 0.01 ^a^

mean ± standard deviation; nd (not detected); DPPH (radical scavenging activity); RP (reducing power); TPC (total polyphenol content); TFC (total flavonoid content); TPAC (total phenolic acid content); TEAC (Trolox equivalent antioxidant capacity); GAE (gallic acid equivalent); QE (quercetin equivalent); CAE (caffeic acid equivalent); different letters in a column denote mean values that statistically differ one from another.

**Table 5:** Specific heat of combustion for Linz biscuit samples.

Sample	*m* (g)	*q*_c_ (kJ/g)	${\bar{q}}_{c}(\mathrm{kJ}/g)$
control	0.706; 0.714	21.898; 22.374	22.136 ^b^
with nettle	0.703; 0.702	22.401; 22.379	22.390 ^a^
with carrot	0.711; 0.707	21.749; 21.703	21.726 ^c^
with elderberry	0.704; 0.703	21.873; 21.552	21.712 ^c^

*m* (mass of sample), ${\bar{q}}_{c}$ (average value); different letters in column denote mean values that statistically differ one from another.

## Data Availability

Written informed consent has been obtained from the sensory evaluators to publish this paper.
